# Ongoing inflammation enhances the toxicity of engineered nanomaterials: Application of an *in vitro* co-culture model of the healthy and inflamed intestine

**DOI:** 10.1016/j.tiv.2019.104738

**Published:** 2020-03

**Authors:** Angela A.M. Kämpfer, Patricia Urbán, Rita La Spina, Isaac Ojea Jiménez, Nilesh Kanase, Vicki Stone, Agnieszka Kinsner-Ovaskainen

**Affiliations:** a European Commission, Joint Research Centre (JRC), Ispra, Italy; b Nano-Safety Research Group, School of Engineering and Physical Sciences, Heriot-Watt University, Edinburgh EH14 4AS, United Kingdom

**Keywords:** Intestinal *in vitro* co-culture, Caco-2, THP-1, Silver nanoparticles, Inflammatory bowel disease

## Abstract

Chronic inflammatory conditions can negatively impact intestinal barrier function and affect the epithelium's interaction with nano-sized materials. We demonstrate the application of a Caco-2/THP-1 co-culture mimicking the intestine in healthy (i.e. stable) or inflamed state in nanotoxicological research. The co-cultures were exposed to non-toxic concentrations of silver nanoparticles (AgNPs) or silver nitrate (AgNO_3_) for 24 h. The barrier integrity and cytokine release as well as necrotic and apoptotic cell death were investigated.

AgNPs and AgNO_3_ most strongly affected the inflamed co-culture. Higher concentrations of AgNPs induced a significant increase in barrier integrity in the inflamed but not the stable co-culture. Necrotic and apoptotic cell death was detected in both conditions but were significantly more pronounced in the inflamed condition. The exposure to AgNO_3_ affected barrier integrity in all experimental set-ups, but caused nuclear condensation only in the Caco-2 monoculture and the inflamed co-culture. AgNPs reduced the release of monocyte chemoattractant protein-1 in the stable model.

Clear differences were observed in the effects of AgNPs and AgNO_3_ in relation to the model's health status. The results suggest an increased vulnerability of the inflamed epithelial barrier towards AgNPs underlining the importance to consider the intestinal health status in the safety assessment of nanomaterials.

## Highlights

• In inflamed conditions, Caco-2 cells are more susceptible to AgNP-induced effects than cells in homeostatic co-cultures.
• Inflammation-like processes enhance the occurrence of AgNP-induced necrosis and apoptosis.
• AgNPs might influence gut homeostasis by inhibiting monocyte chemoattractant protein-1.

## 1 Introduction

The intestine is a semi-permeable barrier that facilitates the uptake of nutrients while at the same time preventing the entry of noxious substances into the organism. In the case of illness – e.g. acute or chronic intestinal inflammation – its functions can be compromised due to the continuous stress induced by pro-inflammatory cytokines, reactive oxygen and nitrogen species (ROS/RNS) as well as thereby caused tissue damage (Konig et al., 2016).

The intestinal epithelial cells (IECs) are continuously in contact with nano-sized structures, like naturally-occurring micelles and proteins or engineered nanomaterials (ENM) that were purposely or accidentally introduced to a food product (Siemer et al., 2018). Even though ample research is conducted to assess the safety of ENM for human health, very little is known regarding the impact of orally ingested ENM in general and in the presence of intestinal inflammatory conditions in particular.

The present study aims to evaluate the applicability of our newly developed *in vitro* model of the human intestine, which can mimic a healthy – i.e. stable – or inflamed state of the intestine (Kämpfer et al., 2017), to study the effects of ENM. For this purpose we used silver nanoparticles (AgNPs), the most abundantly used ENM in consumer products according to the Consumer Products Inventory (2016). AgNPs are most commonly used in medical devices (Roe et al., 2008; Maneerung et al., 2008) and food contact materials (Llorens et al., 2012), due to their bactericidal activity against both Gram-positive and Gram-negative bacteria, including multi-drug resistant strains (Lara et al., 2009; Sondi and Salopek-Sondi, 2004). Their application in consumer products suggests that a wide public might be exposed to AgNPs, which stresses the need to assess their safety.

The impact of AgNP exposure has been extensively studied in a variety of cell types of human and animal origin with a focus on cytotoxic (Sambale et al., 2015; Beer et al., 2012), genotoxic (Li et al., 2012; Foldbjerg et al., 2011) and immuno-modulating effects (Yang et al., 2012; Park et al., 2010). Nevertheless, questions remain due to contradictory results derived from a variety of model types and the use of particles with variable characteristics. Deleterious effects were often demonstrated *in vitro* and to a lesser extent *in vivo*, but the hazard for humans remains unclear. The only available human AgNPs exposure study neither observed a pro-inflammatory response nor indications for (organ) toxicity (Munger et al., 2014). However, this study did not account for prolonged exposure and potential accumulation of AgNPs, which might cause chronic toxicity. Furthermore, the great majority of *in vitro* and *in vivo* hazard studies did not investigate conditions of impaired health like intestinal inflammation, even though multiple studies suggested a different interaction with and uptake of ENM in inflamed compared to healthy intestinal tissue (Hassani et al., 2009; Schmidt et al., 2013; Lamprecht et al., 2001). Despite this clear knowledge gap and the recognised need for disease models (Lefebvre et al., 2015), only few publications have applied biologically relevant *in vitro* models in nanotoxicity studies (Susewind et al., 2016; Leonard et al., 2010).

To address this gap in the literature, we investigated the impact of the intestinal health status at the time of exposure on the cytotoxic, barrier-regulating and immuno-modulating potential of AgNPs using the novel *in vitro* co-culture model of the healthy or inflamed intestine (Kämpfer et al., 2017).

## 2 Materials and Methods

### 2.1 Materials

Accutase, Alcian Blue, β-nicotinamide adenine dinucleotide sodium salt (NAD), Bovine Serum Albumin (BSA), Tri-Sodium citrate dihydrate, interferon gamma (IFN-γ), iodonitrotetrazolium chloride (INT), lithium lactate, lipopolysaccharide (LPS), phenazine methosulfate (PMS), phorbol 12-myristate 13-acetate (PMA), silver nitrate (AgNO_3_), sucrose, tannic acid, 3,3′,5,5′-Tetramethylbenzidine (TMB), Tris-base, Tris-HCl, and Triton-X100 were purchased from Sigma Aldrich (Milan, Italy). Minimum essential medium (MEM), RPMI medium, D-glucose, foetal bovine serum (FBS), L-glutamine, 2-mercaptoethanol (ME), penicillin/streptomycin (pen/strep), and sodium pyruvate were purchased from Thermo Fisher Scientific (Monza, Italy).

### 2.2 AgNP synthesis and characterisation

AgNPs were synthesised by reducing AgNO_3_ with Tri-Sodium citrate dihydrate and tannic acid using a modified procedure of Dadosh (2009). Tannic acid (2 × 10^−3^ M) was added to 6 mL of citrate (28 mM) and stirred at 60 °C for 15 min. Subsequently, 6 mL of the solution were added to 94 mL of 0.55 mM AgNO_3_ in boiling condition under vigorous stirring, and kept at 97 °C for 40 min using a microwave synthesis reactor (Discover S, CEM Corporation). Thereafter, the suspension was rapidly cooled to room temperature (RT). The absence of endotoxin contamination in the particle stock suspension was tested using a commercially available kit (LAL test; Thermo Fisher Scientific).

The particle size distribution of AgNPs (5 μg mL^−1^) was measured using Transmission Electron Microscopy (TEM) and Centrifugal Liquid Sedimentation (CLS) in dispersant as well as after 4 h, 24 h, and 48 h incubation in MEM-based cell culture medium (CCM) (composition below) at standard culture conditions (37 °C, 5% CO_2_).

For TEM imaging, ultrathin Formvar-coated 200-mesh copper grids (Tedpella Inc.) were coated with Alcian Blue as described by Mast and Demeestere (2009). Subsequently, the grids were placed onto a drop of particle stock suspension for 20 min and thoroughly washed with MilliQ H_2_O thereafter. The particles were visualised using a TEM (JEOL 2100, Japan) at an accelerating voltage of 200 kV. For each sample, the size of at least 100 particles was measured to obtain the average diameter and the size distribution. Digital images were analysed with the ImageJ software and a custom macro performing smoothing (3 × 3 or 5 × 5 median filter), manual global threshold and automatic particle analysis provided by ImageJ (macro available on http://code.google.com/p/psa-macro). To exclude agglomerates formed during drying a circularity filter of 0.8 was used. Energy-dispersive X-ray spectroscopic (EDS) analysis was performed with a QUANTAX EDS detector (Bruker, USA) in automatic acquisition mode and with the same background correction. The samples were stored at 4 °C protected from light. The analysis was carried out within 5 days of sample preparation.

CLS measurements were used to determine the particles' hydrodynamic diameter in dispersant and CCM. The disc centrifuge (CPS Instruments, CD24000UHR) was run at 22,000 rpm. A sucrose density gradient was established ranging from 8 to 24% (*w*/w) by successively injecting 9 varying ratios of a low density (2 g in 23 g H_2_O) and a high density (6 g in 19 g H_2_O) sucrose solution. After injecting the lowest concentration, 0.5 mL of dodecane was injected and the gradient was allowed to equilibrate for 30 min. The gradient's quality was confirmed using a reference sample (Sigma-Aldrich, 730,807) after the equilibration and a PVC calibration standard prior to sample measurement. Each sample was injected in a volume of 0.1 mL.

### 2.3 Cell culture

Caco-2 cells (ACC169, DSMZ, Braunschweig, Germany) were cultured in MEM-based CCM supplemented with 20% FBS and 1% pen/strep at standard culture conditions. The THP-1 cells (ATCC, TIB-202) were grown in RPMI-based CCM supplemented with 10% FBS, 1% pen/strep, 1% L-glutamine, 1 nM sodium pyruvate, 0.7% D-glucose, and 0.1% ME at standard culture conditions. Both cell lines were tested for their genetic integrity (DSMZ, Braunschweig, Germany) and showed fully matching STR reference profiles compared to the distributors' profiles. The cell lines tested negatively for mycoplasma contamination by qPCR (Minerva Biolabs GmbH, Germany).

The stable and inflamed co-cultures were established as described earlier (Kämpfer et al., 2017). Briefly, Caco-2 cells (1.8 × 10^5^ cells cm^−2^) were seeded on transparent PET transwell inserts (1 μm pore size; Falcon) and maintained for 18–21 days. On the apical side, the cells were cultured in MEM, whereas the medium in the basolateral compartment was gradually changed to RPMI-based THP-1 medium without ME. At the start of the co-culture, the Caco-2 barriers reached TEER values between 360 and 390 Ω•cm^2^. The THP-1 cells were seeded in 25 cm^2^ flasks (3 × 10^6^ cells per flask) and differentiated with PMA (100 nM) for 24 h. Subsequently, the cells were detached with Accutase, plated on transwell-suitable 12-well plates (1.8 × 10^5^ cells per well), and allowed to re-attach for 1 h. The stable co-culture was started by placing a transwell insert with differentiated Caco-2 cells onto a THP-1-containing well. For the inflamed set-up, the Caco-2 cells were basolaterally primed with IFN-γ (10 ng mL^−1^) for 24 h as described by Wang et al. (2005) before the co-culture. The THP-1 cells were pre-exposed to *E. coli-*derived LPS and IFN-γ (10 ng mL^−1^ each) for 4 h before the co-culture. The stable and inflamed co-cultures were started in parallel.

### 2.4 Exposure to AgNPs and AgNO_3_

*96-well plate*: Caco-2 cells were seeded (1 × 10^4^ cells per well) and maintained for 72 h at standard culture conditions. The AgNPs and AgNO_3_ exposure suspensions/solutions were prepared freshly before each experiment by diluting the stock suspension/solution (0.5 mM and 0.55 mM, respectively) to 0.02–20 μg mL^−1^ in fresh CCM. For the exposure, the cell supernatant was discarded and replenished with 50 μL fresh CCM per well. 50 μL of the AgNP suspension or the AgNO_3_ solution were added with the exposure concentrations eventually ranging from 0.01–10 μg mL^−1^. After 24 or 48 h, the supernatants were collected for the quantification of LDH. Subsequently, the cells were fixed and used for high content analysis (HCA).

*Transwell plates*: The Caco-2 monoculture and co-culture models were set up as described before and allowed to equilibrate for 24 h before being apically exposed to

• 1 or 10 μg mL^−1^ AgNPs or
• AgNO_3_ with an Ag-content equivalent to 1 μg mL^−1^ AgNPs.

**Fig. 1:**
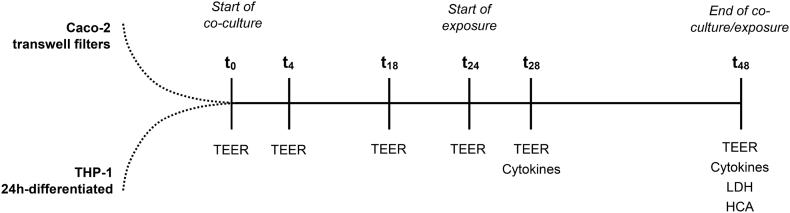
Schematic overview of the experimental time points.

For AgNPs, the exposure suspension was prepared as described above. The AgNO_3_ stock (0.55 mM) was directly added apically to obtain the appropriate exposure concentration. After 24 h of exposure, the cell supernatants were collected for the quantification of LDH and cytokines. The transwells were fixed and used for high content analysis (HCA) (Fig. 1).

After initiation of the co-cultures, the models were sustained for a total of 48 h. Over the first 24 h (at t_0_, t_4_; t_18_, and t_24_) the transepithelial electrical resistance (TEER) was measured to monitor Caco-2 barrier integrity. At t_24_, the cultures were exposed to AgNPs (1 or 10 μg mL^−1^), AgNO_3_ or kept as unexposed control. After 4 h (t_28_), the TEER was measured and supernatant samples were taken from the basolateral and apical side for cytokine analysis. At the end of the 24 h exposure period (t_48_), the TEER was measured before the supernatants from both apical and basolateral compartment were collected for the quantification of cytokines and LDH. Finally, the filter inserts were washed and fixed for analysis using the IN Cell Analyzer.

### 2.5 Measurement of barrier integrity

The TEER was measured using a Voltohmmeter (Millicell, ERS-2) to monitor the barrier integrity throughout the co-culture with THP-1 cells and in response to the exposure to AgNPs and AgNO_3_. For studying the effect of co-culturing Caco-2 and THP-1 cells the TEER was measured just before the co-culture and after 4, 18, 24, 28, and 48 h. In cultures exposed to AgNPs or AgNO_3_ the TEER was measured after 4 and 24 h of exposure (see Fig. 1). Before measurements, the electrode was sterilised (15 min) in 70% ethanol and neutralised in pre-warmed PBS and MEM. The results were corrected for the blank value and multiplied by the filter size to obtain the final results in Ohm x cm^2^ (Ω·cm^2^).

### 2.6 Lactate dehydrogenase (LDH) assay

The LDH assay was used to investigate necrotic cell death after the exposure to AgNPs or AgNO_3_. To measure the LDH activity, 50 μL of 200 mM TRIS (10.6 mg Tris-base and 22.2 mg Tris-HCl in 1 L H_2_O), 50 μL of 50 mM lithium lactate, and 50 μL mix of INT, PMS, and NAD were added to a 96-well plate at concentrations of 1.32 mg mL^−1^, 0.36 mg mL^−1^and 3.44 mg mL^−1^, respectively. A sample of cell-free supernatant (50 μL) was incubated for 5 min at RT. The optical density was measured spectrophotometrically (Perkin Elmer, Enspire) at 490 nm. A background control was subtracted from the results to account for potential LDH activity of FBS. Caco-2 cells exposed to 0.1% Triton-X100 for 24 h served as positive control.

### 2.7 HCA

The IN Cell Analyzer 2200 (GE Healthcare, UK) was used to quantify the number of DAPI-stained nuclei in monocultures of undifferentiated and differentiated Caco-2 cells as well as stable and inflamed co-cultures after exposure to AgNPs or AgNO_3_. The Caco-2 cells were washed with PBS, fixed in 3.7% formaldehyde (15 min), and stained with DAPI (1:4000) (15 min) at standard culture conditions. The transwell filters were cut from the plastic supports and mounted onto standard microscopy slides cells facing up.

*96-well plates*: A minimum of 12 fields was imaged per well using a 20× objective. The data analysis was performed on the IN Cell Investigator Software (GE Healthcare, UK) using in-house developed protocols with a minimum of 10,000 cells in the negative control. The cell viability was calculated as percentage of nuclei in the exposure conditions compared to the negative control.

*Transwell plates*: A minimum of 12 fields per filter were imaged using a 60× objective. To obtain a higher resolution, images were acquired as a z-stack of three images (1 μm each), and converted into a 2D image using the IN Cell Investigator Software. The data analysis was performed using the IN Cell Investigator Software and in-house developed protocols.

### 2.8 Cytokine quantification

*ELISA:* After 24 h exposure to AgNPs or AgNO_3_, the release of IL-1β, IL-8, and TNF-α was quantified in cell-free supernatants by sandwich ELISA as previously described (Kinsner et al., 2006). Briefly, the primary antibody (R&D, DY210/201/208) was incubated in coating buffer (0.1 M NaHCO_3_ in MilliQ H_2_O) on high protein-binding 96-well plates (Nunc MaxiSorp). After washing (0.05% Tween-20 in PBS) and blocking (3% BSA/PBS) the samples were added pure or diluted (1:2–1:10 in 1% BSA/PBS) and incubated for 2 h. After incubation (30 min) with peroxidase (Bio Trend), 100 μL TMB were added and the reaction stopped with sulphuric acid (1 M). The absorbance was read spectrophotometrically at 450 nm. A calibration curve (7 concentrations in duplicates) and blanks were included. The calibration curve was plotted as 4-parameter logfit.

*Bio-Plex MAGPIX:* The release of MCP-1, MIP-1α, IFN-γ, IL-4, IL-6, and TNF-α were quantified using a magnetic bead-based assay analysed with the Bio-Plex MAGPIX Multiplex Reader. For the analysis, supernatants were taken after 4 h of exposure to AgNPs or AgNO_3_.

The magnetic anti-cytokine beads (Bio-Rad, 171B50–04, 06, 19, 21, 22, 26) were diluted 12.5-times in Bio-Plex Assay Buffer and a master mix was prepared. The master mix (25 μL) was transferred to each well before addition of samples, standards or the blank. Monoculture and stable co-culture supernatants (50 μL) were added and the plate was incubated for 30 min at RT. The supernatant samples from inflamed co-cultures were diluted 1:2 in diluent buffer. After washing, the detection antibody master mix was added and the plate incubated for 30 min at RT. After washing, the wells were incubated with streptavidin-PE (1:100 diluted in Assay Buffer) and incubated for 10 min at RT. All wells were washed and the beads re-suspended in 150 μL Assay Buffer for the read-out (Bio-Plex MAGPIX, Bio-Rad). For each plate a standard curve (8 concentrations plated in duplicate) and two blanks were included. The calibration curve was plotted as 4 parameter logfit.

### 2.9 Statistical analysis

The data analysis was performed with Microsoft Excel. The results are presented as average of at least three independent experimental runs (*N* = 3) with 3 replicates each unless stated otherwise. The results were illustrated using GraphPad Prism 6. Variations between results are expressed as standard deviation (SD). The statistical analysis was done by one-way ANOVA and Dunnett's test, or if applicable unpaired *t*-test. A value of *p* ≤ 0.05 was accepted as statistically significant. No symbolic distinction is made between p ≤ 0.05, ≤ 0.01, ≤ 0.005, and ≤ 0.001 in graphs.

## 3 Results

### 3.1 AgNPs characterisation

**Fig. 2:**
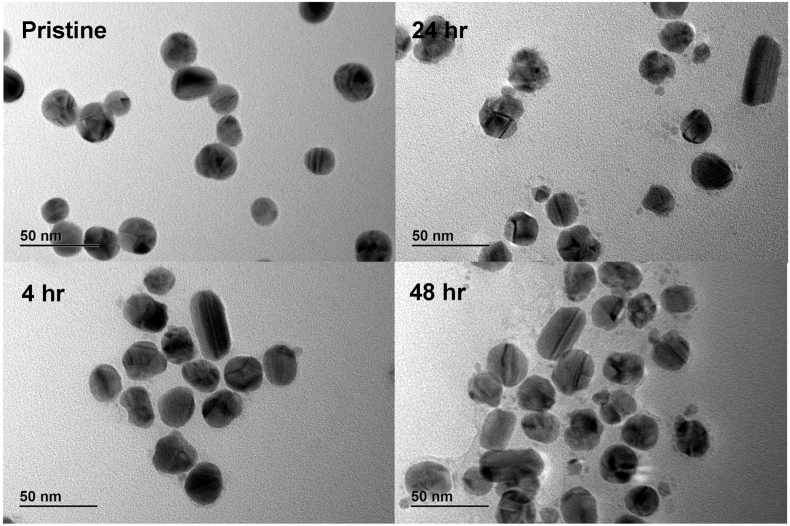
TEM imaging of AgNPs in dispersant (pristine) and after 4, 24, and 48 h incubation in CCM at standard culture conditions.

The AgNPs characterisation results are summarised in Table 1. According to TEM analysis, the mean primary particle diameter was 23 ± 4.2 nm in dispersant. The AgNPs were foremost of spherical shape with low occurrence of rod-shaped particles (Fig. 2). After incubation in CCM, both TEM images and their quantitative analysis (Fig. S1) suggested a continuous dissolution of the particles, which was paralleled by an increasing number of smaller-sized particles of ~2.5 nm. Nevertheless, the average diameter remained at ~23 nm over the first 24 h and reduced to 19.1 nm after 48 h.

**Table 1:** Characterisation of AgNPs in dispersant and CCM.

Instrument & parameter		Dispersant	CCM		
			4 h	24 h	48 h
TEM	Size (nm)	23.0 ± 4.2	23.5 ± 6.1	23.7 ± 7.5	19.1 ± 8.3
	Shape	Spherical, few rods	Increasing number of smaller particles		
CLS	Size (nm)	23.6 ± 1.3	18.4	19.7	18.3
	HHW^a^ (nm)	4.1	5	5	4.9

a HHW = Half Height Width.

CLS measurements of AgNPs in dispersant showed a hydrodynamic diameter of 23.6 ± 1.3 nm, which decreased to 19.7 nm and 18.3 nm after 24 h and 48 h incubation in CCM at standard culture conditions, respectively (Fig. S2). It should be noted, that the hydrodynamic diameter calculations by CLS are based on the density of bulk Ag (i.e. 1.05 g mL^−1^). However, both tannic acid and a protein corona could influence the particle density and, hence, cause an overestimation of the diameter calculations (Capomaccio et al., 2015).

### 3.2 Ag-induced cytotoxicity in undifferentiated Caco-2 cells

The cytotoxic potential of AgNPs and AgNO_3_ was studied in undifferentiated, sub-confluent Caco-2 cells by quantification of LDH activity and HCA of DAPI-stained nuclei.

**Fig. 3:**
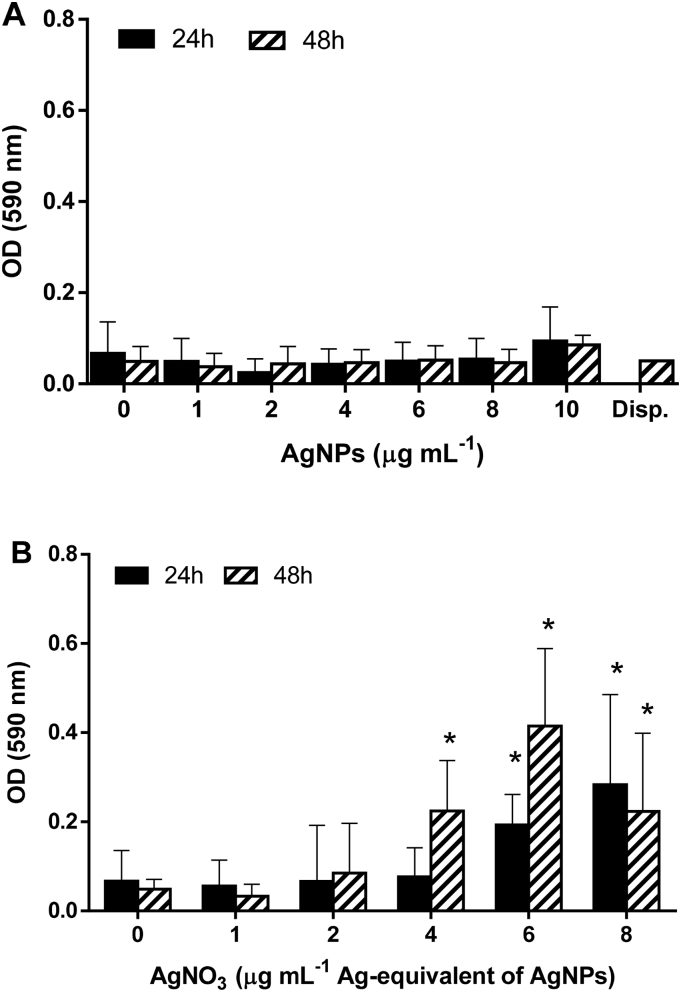
Caco-2 LDH release after 24 h and 48 h exposure to (A) AgNPs and (B) AgNO_3_. (Average ± SD, *N* = 3; *p ≤ 0.05 compared to control; Disp. = dispersant control).

After 24 and 48 h exposure to AgNPs, no significant increase in LDH activity was detected in Caco-2 supernatants for concentrations up to 10 μg mL^−1^ or for the dispersant control (Fig. 3A).

In AgNO_3_-exposed undifferentiated Caco-2 cells, a significantly increased LDH activity was measured after 24 h exposure to concentrations ≥6 μg mL^−1^ AgNPs (Fig. 3B, black bars). After 48 h, already concentrations ≥4 μg mL^−1^ AgNPs induced significant increases in LDH activity (Fig. 3B, striped bars).

**Fig. 4:**
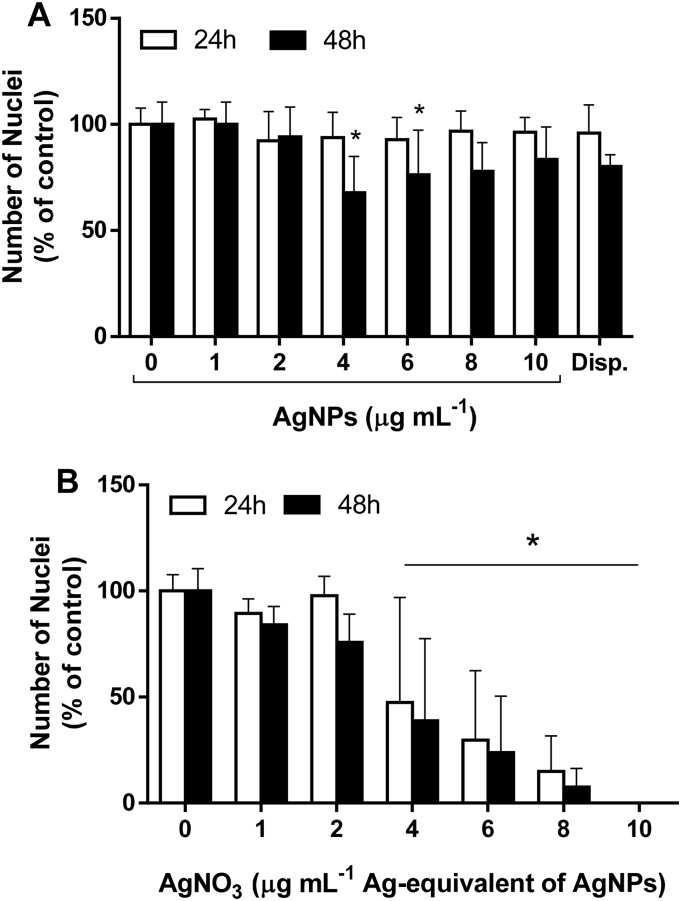
Quantification of DAPI-stained nuclei after 24 and 48 h exposure to (A) AgNPs and (B) AgNO_3_ (Average ± SD, *N* = 2, Dispersant (i.e. ‘Disp.’) and AgNO_3_ 10 μg mL^−1^: *N* = 1 *p ≤ 0.05 compared to control).

The analysis of DAPI-stained nuclei confirmed that a 24 h exposure of up to 10 μg mL^−1^ AgNPs did not induce a significant reduction in cell number compared to the negative control (Fig. 4A). Interestingly, the cell numbers were significantly reduced for AgNPs exposure concentrations of 4 and 6 μg mL^−1^ after 48 h (*p* = 0.003 and *p* = 0.044, respectively). At both concentrations of <4 and > 6 μg mL^−1^ AgNPs no significant differences were detected.

In AgNO_3_-treated Caco-2 cells, the cell numbers reduced drastically in a dose-dependent manner starting from exposure concentrations equivalent to 4 μg mL^−1^ AgNPs (Fig. 3B). The cell numbers were slightly but not significantly lower after 48 h compared to 24 h of exposure. At the highest exposure concentration virtually no stained cells were detected.

### 3.3 Effect of AgNP- and AgNO_3_-exposure on barrier integrity

**Fig. 5:**
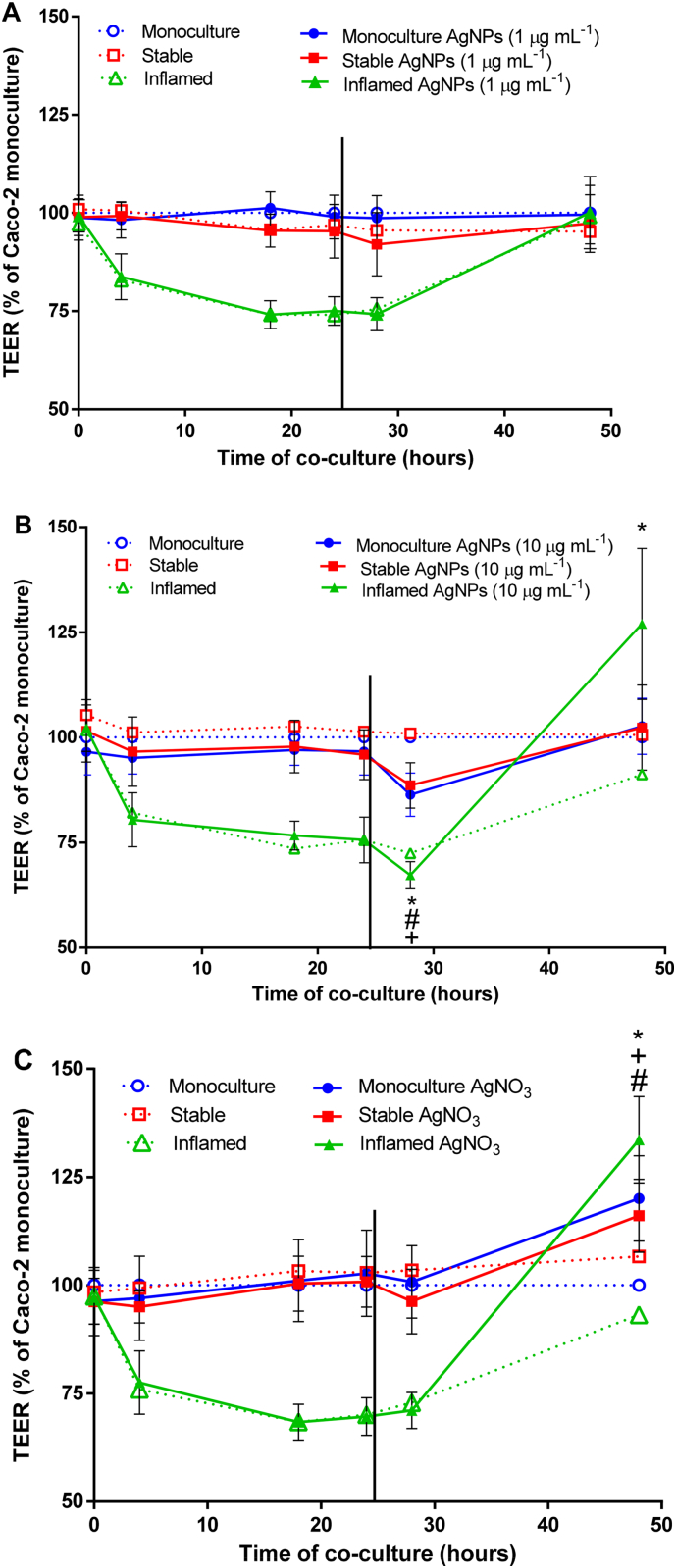
Barrier integrity measured as TEER over 48 h of co-culture and 24 h exposure to AgNPs or AgNO_3_.

The effects of AgNP- and AgNO_3_-exposure on barrier integrity were assessed in three Caco-2 culture models: monoculture, stable co-culture and inflamed co-culture. The characteristics of both co-culture models were in line with the previously defined requirements (Kämpfer et al., 2017) before exposure experiments were conducted. The TEER of the unexposed stable co-culture did not differ significantly from the Caco-2 monoculture control at any of the investigated time points (Fig. 5A-C). In contrast, after initiation of the inflamed co-culture the TEER decreased rapidly by ≥25% and remained significantly reduced (*p* ≤ 0.001) compared to the monoculture until 28 h of co-culture (Fig. 5A-C). After 48 h, the TEER of the inflamed co-culture was fully re-established.

As presented in Fig. 5A, the lower concentration of 1 μg mL^−1^ AgNPs did not affect the TEER over an exposure period of 24 h in any of the three culture models.

For the higher concentration of 10 μg mL^−1^ AgNPs (Fig. 5B), a significant decrease (*p* ≤ 0.003) in TEER by 8–14% was measured in all three culture models after 4 h of exposure (t_28_). After 24 h of exposure (t_48_), the TEER of the Caco-2 monoculture and stable co-culture recovered to the values of to their respective controls. In contrast, the TEER of the inflamed co-culture was significantly increased (127 ± 17.9%, p ≤ 0.001) compared to the Caco-2 monoculture control.

AgNO_3_ was included as an ion control (Fig. 5C). The three culture models were exposed to an Ag-equivalent of 1 μg mL^−1^ AgNPs. The short-term exposure of 4 h did not induce any changes in TEER. Following 24 h of exposure, the TEER was significantly increased (p ≤ 0.003) in all three culture conditions. The effect was most pronounced in the inflamed co-culture with a TEER of 133 ± 9.9% compared to the Caco-2 monoculture control. In the Caco-2 monoculture and stable co-culture, the TEER increased to 120 ± 9.6% and 116 ± 8.4%, respectively.

24 h after initiation of the co-cultures (indicated by the black line), Caco-2 monocultures, stable and inflamed co-cultures were exposed to (A) 1 μg mL^−1^ AgNPs, (B) 10 μg mL^−1^ AgNPs or (C) AgNO_3_ (Ag-equivalent to 1 μg mL^−1^ AgNPs) for 24 h. For each of the three culture models an unexposed control was included (dotted lines). The TEER was expressed as percentage of the unexposed Caco-2 monoculture control. Exposure to 10 μg mL^−1^ AgNPs caused a significant increase in TEER in the inflamed co-culture after 24 h. AgNO_3_ treatment induced a significant TEER increase in all three culture models. For reasons of practicality only the SD of the exposed cultures are shown. The SD of the control conditions were within 3.9–9.2% (monoculture), 2.8–10.3% (stable co-culture), and 2.3–7.6% (inflamed co-culture) (average ± SD, *N* ≥ 3, *p ≤ 0.05 monoculture compared to corresponding control; ^#^p ≤ 0.05 stable co-culture compared to corresponding control, ^+^p ≤ 0.05 inflamed co-culture compared to corresponding control).

### 3.4 LDH release in transwell cultures

**Fig. 6:**
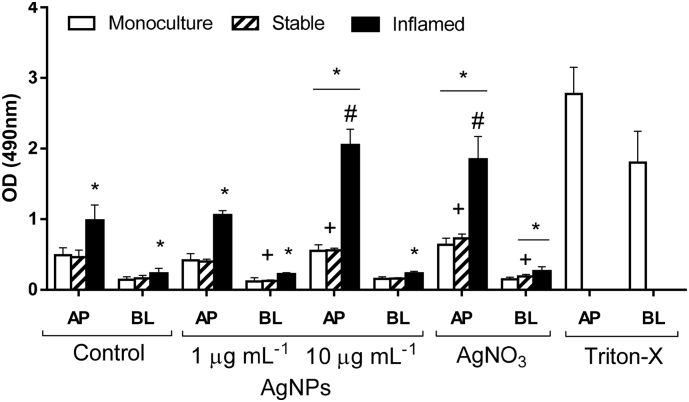
LDH release in Caco-2 monoculture, stable, and inflamed co-cultures after exposure to AgNPs or AgNO_3_.

The LDH activity was determined in apical and basolateral supernatants after 48 h of co-culture including 24 h exposure to AgNPs or AgNO_3_ (Fig. 6). An unexposed control was included for each culture condition. No significant differences in LDH activity were detected between the unexposed Caco-2 monoculture (white bars) and stable co-culture control (striped bars). The LDH activity in supernatants of the inflamed co-culture (black bars) doubled to 200% and 213% in the apical and basolateral compartment, respectively.

The exposure to 1 μg mL^−1^ AgNPs did not cause significant changes in LDH release in any of the culture conditions compared to their respective controls. After the exposure to 10 μg mL^−1^, the apical LDH activity was significantly increased in all three culture models compared to both the monoculture control and their respective untreated controls. The effect was most pronounced in the inflamed model with an increase of 210% after exposure to 10 μg mL^−1^ AgNPs compared to its untreated control. In the monoculture and stable co-culture the increase was moderate with 112% and 121% compared to the corresponding negative control, respectively. No changes were observed in basolateral samples.

Also AgNO_3_ caused a significant increase in apical LDH release in all three culture models. Again, the effect was most pronounced in the inflamed co-culture with 190% compared to the unexposed inflamed co-culture. In the monoculture and stable co-culture, the apical LDH release was moderately increased by 130% and 160% compared to the corresponding controls. In the stable co-culture, also the LDH activity in basolateral supernatants was increased slightly but significantly by 120%.

The cultures were exposed to AgNPs (1 or 10 μg mL^−1^) or AgNO_3_ (Ag-equivalent to 1 μg mL^−1^ AgNPs) 24 h after initiation of the co-culture. Following 24 h of exposure, the LDH release was significantly elevated in the inflamed co-culture compared to the monoculture and stable co-culture controls. The exposure to 1 μg mL^−1^ AgNPs did not alter the LDH activity, but treatment with 10 μg mL^−1^ AgNPs significantly increased LDH activity. Also the exposure to AgNO_3_ increased the LDH release in all three culture conditions. Cultures exposed to 0.1% Triton X-100 for 24 h served as positive control. (Average ± SD, *N* ≥ 3; *p ≤ 0.05 compared to monoculture control, ^+^p ≤ 0.05 compared to stable co-culture control; ^#^p ≤ 0.05 compared to inflamed co-culture control).

### 3.5 Morphology and integrity of Caco-2 nuclei

**Fig. 7:**
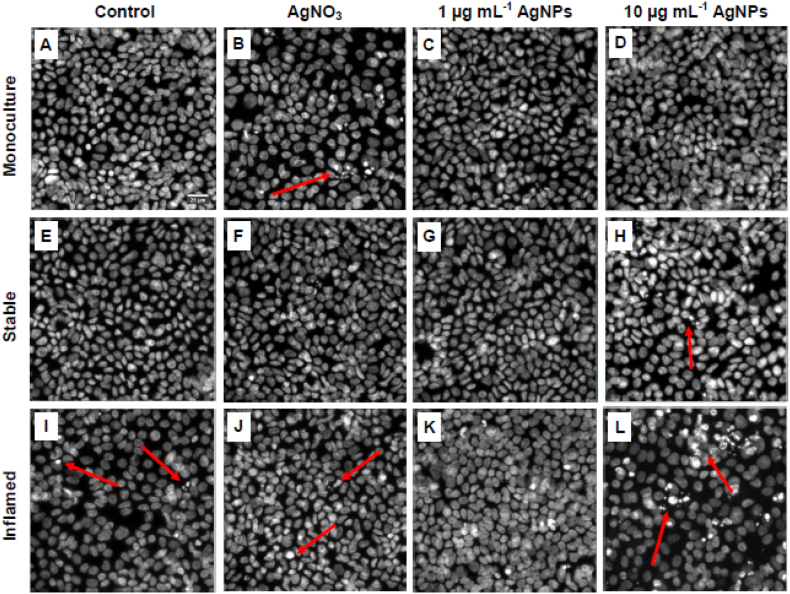
DAPI-stained Caco-2 nuclei after 24 h exposure to AgNPs or AgNO_3_.

At t_48_, after 24 h of exposure, the Caco-2 cell layers of all culture models and exposure conditions were stained with DAPI and imaged using the IN Cell Analyzer. Representative example images of all conditions are presented in Fig. 7.

In the unexposed conditions, the Caco-2 cells form a dense and continuous monolayer (Fig. 7A, E, and I). In the inflamed co-culture control (I) a number of fragmented nuclei is visible.

The exposure to AgNO_3_ appeared to have reduced the number of nuclei in the Caco-2 monocultures and increased the occurrence of fragmented nuclei (B). Both assumptions were confirmed by a quantitative analysis of one experiment (Fig. S3). No changes in the distribution and integrity of nuclei were noted in AgNO_3_-exposed Caco-2 barriers of the stable co-culture (F) compared to its respective control. In inflamed co-cultures (J), AgNO_3_ caused an increased occurrence of fragmented nuclei. No changes in the number or integrity of nuclei were observed after the exposure to 1 μg mL^−1^ AgNPs in the monoculture (C) or stable co-culture (G). These results are supported by the quantitative image analysis (Fig. S3). The nuclear surface in the inflamed co-culture appeared to be increased (K) but was not quantified.

Most pronounced effects were observed after exposure to 10 μg mL^−1^ AgNPs. The distribution of nuclei appeared ragged and the number of fragmented nuclei was markedly increased (L). Similar trends were observed in the stable model (H), and to a lesser extend in the Caco-2 monoculture (D) (Fig. S3).

(A-D) Caco-2 monoculture, (E-H) stable co-culture, and (I-L) inflamed co-culture were maintained for 48 h in CCM (Control) or for 24 h with subsequent 24 h exposure to AgNPs (1 or 10 μg mL^−1^) or AgNO_3_ (Ag-equivalent to 1 μg mL^−1^ AgNPs). The cell cultures were apically exposed and after 24 h incubation washed, fixed, DAPI-stained and imaged with the IN Cell Analyzer (60× magnification, scale bar = 25 μm). Red arrows indicate fragmented nuclei.

### 3.6 Cytokine release at t_28_

After 4 h exposure, the levels of TNF-α, IFN-γ, IL-6, and IL-4 in supernatants of the Caco-2 monoculture remained below the detection limit of the assay (Fig. S4A). Except for two samples, the concentrations remained close to the unexposed control. The exposure to 1 and 10 μg mL^−1^ AgNPs induced a non-significant increase in the levels of MCP-1 to 330 ± 655 and 223 ± 184% of the control.

In the stable co-culture (Fig. S4B), TNF-α, IFN-γ, IL-6, and IL-4 remained below the detection limit. After the exposure to 10 μg mL^−1^ AgNPs, both MIP-1α and MCP-1 were reduced to 21 ± 17 and 44 ± 33% of the unexposed control. For MCP-1 the reduction was statistically significant (*p* = 0.033).

In contrast, high concentrations of all cytokines except IL-4 were detected in the exposed inflamed co-cultures (Fig. S3C). However, neither the exposure to AgNPs nor AgNO_3_ caused significant changes compared to the unexposed inflamed co-culture.

### 3.7 Cytokine release at t_48_

**Fig. 8:**
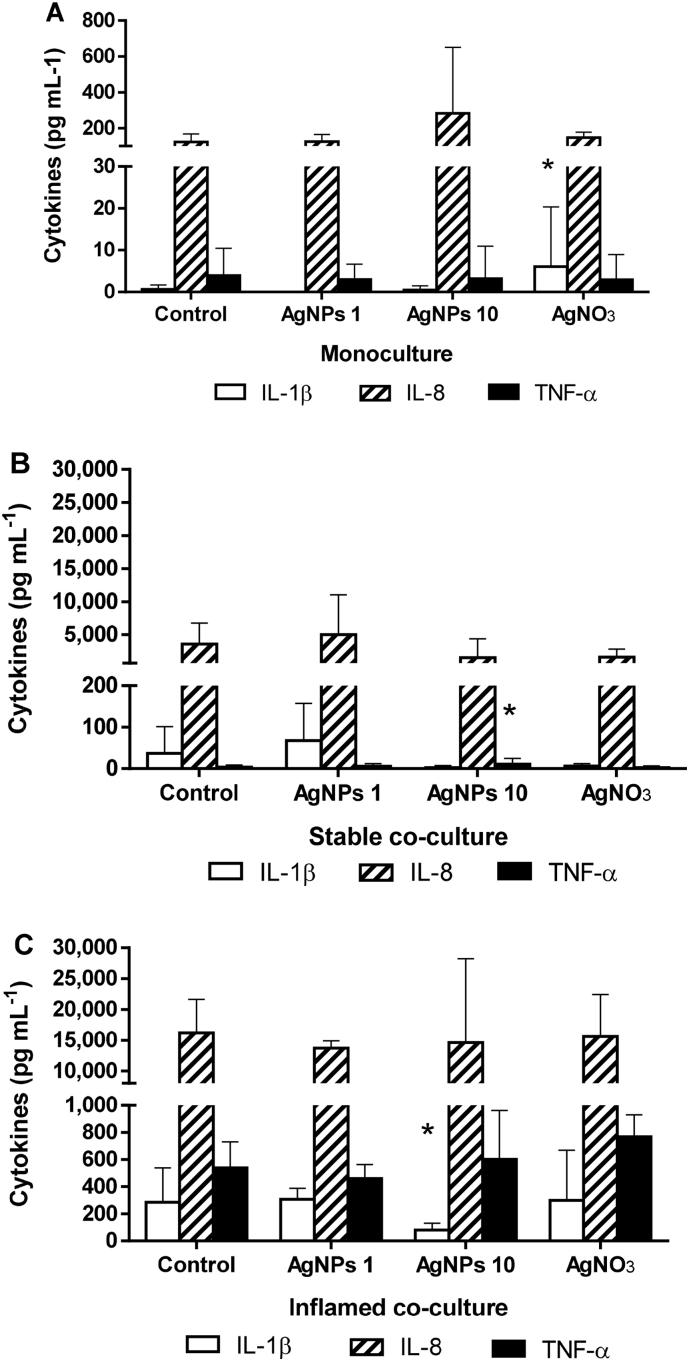
Cytokine release in response to 24 h exposure to AgNPs or AgNO_3_.

After 48 h of co-culture, a clear difference in the release of IL-1β, IL-8, and TNF-α was detected between the controls of the three culture models (Fig. 8A-C). In the Caco-2 monoculture, IL-1β and TNF-α were expressed at concentrations close to or below the detection limit (0.6 ± 1.02 and 3.9 ± 6.4 pg mL^−1^, respectively) (Fig. 8A). Low concentrations of IL-8 were detected (123 ± 43.7 pg mL^−1^).

In the stable co-culture, slightly higher concentrations of IL-1β were measured (37.1 ± 64 pg mL^−1^) (Fig. 8B), whereas TNF-α concentrations remained low to undetectable (3.9 ± 4.5 pg mL^−1^). The release of IL-8 was markedly increased reaching ~3500 pg mL^−1^.

All three investigated cytokines were strongly increased in supernatants of the inflamed co-culture (IL-1β: 286 ± 254 pg mL^−1^, IL-8: 16,190 ± 5442 pg mL^−1^, TNF-α: 539 ± 190 pg mL^−1^) (Fig. 8C).

The exposure to AgNPs or AgNO_3_ for 24 h did not cause systematic changes in cytokine release in any of the culture conditions. No effects were observed after exposure to 1 μg mL^−1^ AgNPs. The exposure to 10 μg mL^−1^ AgNPs induced a slight but significant increase (*p* = 0.027) in TNF-α in the stable co-culture (11.1 ± 13.5 pg mL^−1^) and a decrease (*p* = 0.05) of IL-1β in the inflamed co-culture (80.1 ± 50.8 pg mL^−1^). In the Caco-2 monoculture, the exposure to AgNO_3_ equivalent to 1 μg mL^−1^ resulted in a significant (*p* = 0.048) increase in IL-1β to 6.09 ± 14.2 pg mL^−1^. In both the stable and inflamed co-culture, AgNO_3_ did not cause changes in the release of the investigated cytokines.

The release of IL-1β, IL-8, and TNF-α was measured after 24 h exposure to AgNPs (1 or 10 μg mL^−1^) or AgNO_3_ (Ag-equivalent to 1 μg mL^−1^ AgNPs) in the basolateral supernatants of (A) Caco-2 monocultures, (B) stable co-cultures, and (C) inflamed co-cultures. The cytokine release between the unexposed monoculture and unexposed stable co-culture controls did not differ significantly. In contrast, the release of all three cytokines was strongly increased in the supernatants of the inflamed co-culture control. The exposure to either AgNPs or AgNO_3_ did not result in systematic changes in any of the culture conditions. (Average ± SD, *N* ≥ 3; *p ≤ 0.05 compared to corresponding control).

## 4 Discussion

The study's aim was to investigate a potential impact of the intestinal health status on the effects of non-toxic concentrations of ENM using our novel *in vitro* co-culture model (Kämpfer et al., 2017) and AgNPs as model particle. The results demonstrated clear differences in the sensitivity of Caco-2 cells to AgNPs and AgNO_3_ in relation to the status of the culture with a significantly increased susceptibility under inflammation-like conditions.

As environmental factors like proteins and salts, but also temperature and extended storage can affect the colloidal stability and integrity of ENM (Teeguarden et al., 2007; Kittler et al., 2010), a thorough characterisation of the AgNPs under experimental conditions was performed. The particles showed a homogenous and narrow size distribution in dispersant. However, the extended incubation time in CCM caused a progressive dissolution, resulting in the formation of smaller particles with a diameter < 10 nm. Whether these objects were remnants of incompletely dissolved AgNPs or formed by re-precipitated Ag ions (Ag^+^) is unclear. It is, therefore, likely that the cells were not only in contact with 23 nm AgNPs, but also significantly smaller nano-sized objects as well as Ag^+^. To distinguish potential particle-specific from ionic effects, an Ag^+^ control was included. The ion release from AgNPs could not be quantified in this project, however, the characterisation data allowed an estimation of the dissolution magnitude. Therefore, an Ag-equivalent of 1 μg mL^−1^ AgNPs was chosen to represent a reasonable scenario of 10% AgNPs dissolution in the highest tested concentration over 24 h. Neither we nor others (Oberemm et al., 2016) observed cytotoxic effects in undifferentiated Caco-2 cells at such low Ag^+^ concentrations.

The induction of cytotoxicity is one of the most commonly reported effects of AgNPs. However, large variations regarding the minimum effective dose prevail in relation to the differentiation status and particle characteristics. For undifferentiated Caco-2 cells, concentrations of 15 μg mL^−1^ < 100 nm AgNPs and 25 μg mL^−1^ of 7 nm AgNPs were found to reduce cell viability by 50 and 80%, respectively (Aueviriyavit et al., 2014; Böhmert et al., 2014). In contrast, Susewind et al. (2016) reported an EC_50_ of 85 μg cm^−2^ AgNPs (<20 nm) in differentiated Caco-2 cells, whereas Georgantzopoulou et al. (2016) did not observe any cytotoxicity up to 100 μg mL^−1^ in differentiated Caco-2 monocultures and Caco-2/HT29-MTX co-cultures for 20 and 200 nm AgNPs. Whereas the mucus released by HT29-MTX cells might result in an increased robustness of the cell culture, these discrepancies underline the importance of particle size in the toxicity assessment. Vila et al. (2018) showed absence of cytotoxicity for concentrations up to 50 μg mL^−1^ AgNPs (8 nm) in differentiated Caco-2 cultures, whereas an EC_50_ of 12.23 μg mL^−1^ was calculated for undifferentiated cells.

As we also investigated (pro-)inflammatory markers in response to AgNPs and Ag^+^ the application of non-toxic exposure concentrations was crucial. In our experiments both the LDH assay and HCA of DAPI-stained nuclei showed that concentrations of 1–10 μg mL^−1^ AgNPs did not induce cytotoxic effects in undifferentiated, sub-confluent Caco-2 cells. Since the vulnerability of adenocarcinoma cell lines, including Caco-2, was previously demonstrated to be inversely related to the differentiation status (Pelletier et al., 1990; Böhmert et al., 2014; Vila et al., 2018), these concentrations were used as ‘non-toxic’ in the co-culture exposure studies. Furthermore, both assays confirmed a higher cytotoxic potential of Ag^+^ compared to AgNPs as was previously demonstrated (Oberemm et al., 2016; Georgantzopoulou et al., 2016).

The decrease in TEER in the inflamed co-culture raised the question, if barrier translocation of AgNPs might be increased and allowing for a direct interaction of AgNPs with the THP-1 cells. Both Vila et al. (2018) and Saez-Tenorio et al. (2019) reported that even smaller AgNPs (<10 nm) did not pass Caco-2 barriers over 24 h although cellular uptake occurred. In an M-cell model, where barrier passage was expected to be increased, the basolateral concentration was detectable but remained very low at ~0.5% of the administered dose (Bouwmeester et al., 2011). Importantly, Bouwmeester and colleagues did not observe differences in translocation between particular and ionic Ag. However, these studies were conducted in models of healthy, intact tissue. We have demonstrated earlier (Kämpfer et al., 2017) that the inflammation-like status did not result in an increased translocation of Lucifer Yellow compared to the stable co-culture or Caco-2 monocultures. Also the results from an IL-1β-activated Caco-2 monoculture showed no increased translocation or *co*-localisation of polystyrene particles with tight junction proteins (Leonard et al., 2010). Therefore, we concluded that the translocation of AgNPs is minimal, if not absent, irrespective of the model condition. The reported effects can reasonably be attributed to the interaction of AgNPs or Ag^+^ with the epithelial barrier rather than the THP-1 cells.

In the transwell cultures, dose-dependent effects of AgNPs on the barrier integrity were observed. Whereas no changes were detected in response to low concentrations, concentrations of 10 μg mL^−1^ resulted in a significant increase in TEER in the inflamed co-culture. Interestingly, the Ag^+^ control induced a TEER increase in all three culture models. Bouwmeester et al. (2011) reported a similar increase in barrier resistance (120–130%) in a Caco-2 M-cell model after the exposure to similarly sized and dosed AgNPs and Ag^+^, but the group did not further discuss this observation. These results are in clear contrast to other studies. Susewind et al. (2016) exposed a model of Caco-2, THP-1, and MUTZ-6 cells in non-inflamed or inflamed state. The group observed the highest sensitivity to PVP-capped AgNPs (<20 nm) in the Caco-2 monoculture with a significant TEER reduction (~20%) at exposure concentrations ≥78.1 μg cm^−2^. Both the non-inflamed and inflamed co-cultures were more resilient with barrier disruption starting from 312.5 and 156.25 μg cm^−2^ AgNPs, respectively (Susewind et al., 2016). Georgantzopoulou et al. (2016) studied the effects of AgNPs (20 and 200 nm) and Ag^+^ in Caco-2/TC7 monocultures and Caco-2/HT29-MTX co-cultures. The group did not observe any effect on the barrier integrity for comparable concentrations of either size of AgNPs or Ag^+^ regardless of the presence of goblet cells. Also Vila et al. (2018) did not find negative effects of 8 nm AgNPs on the Caco-2 barrier integrity. However, the group reported an upregulation of tight junction-associated proteins at concentrations ≥10 μg mL^−1^. Our attempt to gain more information on the tight junction integrity by immunocytochemical staining generated ambiguous results (data not shown).

The increase in TEER was unlikely caused by the physical properties of Ag since the effect was only detected after prolonged exposure to AgNPs or Ag^+^ and not in all culture models equally. Instead, we suggest the increase might be related to changes in the Caco-2 barrier. When monitoring cell barrier formation, the increase in TEER corresponds to the cell density and the increasing restriction of the paracellular ion transport by tight junctions (Anderson and Van Itallie, 2009). In a fully developed barrier, an increase in TEER might indicate a further tightening of the barrier, e.g. due to an over-expression of tight junction proteins (Yan and Ajuwon, 2017) or a reduction of intra- and intercellular space as a result of cell swelling (Olsson et al., 2006), the latter of which can be a sign of necrotic cell death (Bonfoco et al., 1995).

The results from the LDH assay indicated the occurrence of necrotic cell death, even though the applied concentrations tested negative in undifferentiated Caco-2 cells. This effect was again most pronounced in the inflamed co-culture, suggesting that stressed Caco-2 barriers were more susceptible to Ag-exposure. These results contradict studies showing an increased resilience of Caco-2 cells after differentiation (Gerloff et al., 2013; McCracken et al., 2015; Vila et al., 2018) as well as the co-culture results by Susewind et al. (2016) and Georgantzopoulou et al. (2016). Both groups reported Caco-2 monocultures to be more sensitive to the exposure to AgNPs than co-cultures with goblet cells (Georgantzopoulou et al., 2016) or immune cells (Susewind et al., 2016). Neither group observed adverse effects after exposure to AgNPs concentrations comparable to ours. Also the exposure to similar concentrations of Ag^+^ was not found to induce cytotoxicity by other groups in Caco-2 monocultures or in co-cultures with goblet cells and after M-cell induction (Oberemm et al., 2016; Georgantzopoulou et al., 2016; Bouwmeester et al., 2011). However, a similar observation of increased sensitivity following differentiation was reported by Thit et al. (2013) regarding the exposure of epithelial cells to copper oxide NPs.

Since the LDH assay can only provide indicative results when used in combination with AgNPs, HCA of DAPI-stained nuclei was used to confirm the occurrence of cell death. The cellular effects were most striking in the inflamed co-culture exposed to Ag^+^ and 10 μg mL^−1^ AgNPs with reduced cell numbers and frequent presence of condensed or fragmented nuclei. The fluorescent intensity (FI), as well as the mean size of the nucleus are important indicators for cellular health and integrity. As the condensation of chromatin and nuclear fragmentation are late events in apoptotic cell death (Tounekti et al., 1995; Collins et al., 1997), both parameters can be used for the identification of apoptotic events. The cause for the observed apoptotic events remains unclear. As previously discussed (Kämpfer et al., 2017), numerous pro-inflammatory cytokines detected in the inflamed co-culture have been reported to induce apoptosis. It is, however, unclear whether the very low increases in TNF-α and/or IL-1β could be responsible for the observed increase in apoptotic events in Caco-2 monocultures and stable co-cultures after exposure to 10 μg mL^−1^ AgNPs and Ag^+^. Instead, AgNPs and Ag^+^ might have affected the cell layers directly. AgNPs have been shown to induce apoptosis in various cell types (Lee et al., 2011; Xue et al., 2016), but not in Caco-2 cells in differentiated or undifferentiated state (Georgantzopoulou et al., 2016; Böhmert et al., 2015; Böhmert et al., 2012). Bouwmeester et al. (2011), however, reported the up-regulation of apoptosis-related genes in a Caco-2-based M-cell model. Similarly, Ag^+^ was found to up-regulate several apoptosis-associated proteins, even though most lacked statistical significance (Georgantzopoulou et al., 2016).

We were unable to tell, if the observed necrotic and apoptotic events were depending on each other or caused by independent pathways. Usually the induction of apoptosis would not result in elevated LDH activity (Chan et al., 2013), which might indicate the occurrence of an alternative cell death mechanism, e.g. secondary necrosis or necroptosis. Under homeostatic conditions, apoptotic cells are quickly cleared to prevent the induction of inflammation (Günther and Seyfert, 2018). A crucial factor for this is the presence of phagocytising cells, e.g. macrophages (Erwig and Henson, 2008). In case the phagocytising capacities are exceeded, apoptotic cells can proceed to undergo secondary necrosis, which features similar characteristics as necrosis (Vanden Berghe et al., 2010). Although the THP-1 cells are present in the model, the spatial separation makes and interaction with the epithelial cells impossible in this culture set-up. Neighbouring epithelial cells are capable of acting as ‘non-professional’ phagocytes (Günther and Seyfert, 2018), but the relevance and capacity of epithelial cells in this process remains unclear. In *in vitro* research, the occurrence of secondary necrosis is generally regarded as artefact.

Necroptosis is a mechanism of controlled cell death that occurs when apoptotic mechanisms are blocked (Vanden Berghe et al., 2010; Negroni et al., 2015). Necroptosis was shown to be active in humans with inflammatory bowel disease, where Caspase-8 was reduced and RIP3, thought to be a key mediator in necroptotic processes, increased (Pierdomenico et al., 2014). Both TNF-α and IFN-γ have been shown to induce necroptosis *in vitro* (Vanden Berghe et al., 2010; Chen et al., 2019). However, the presence of RIP3 expression in Caco-2 cells remains elusive. Whereas He et al. (2017) reported its expression to be absent, Lou et al. (2019) observed ample expression under normal conditions as well as upregulation in response to TNF-α.

Finally, our results do not indicate a clear pro- or anti-inflammatory potential of AgNPs as no systematic changes in the cytokine profile were detected in either culture model. Several *in vivo* studies reported anti-inflammatory effects of AgNPs (Hebeish et al., 2014; Wong et al., 2009), however, not following oral exposure. The majority of identified oral exposure studies observed either pro-inflammatory effects, e.g. through the release of pro-inflammatory cytokines (Greulich et al., 2011) and impacting the distribution of immune cells (Park et al., 2010) or no immune-modulating effects at all (van der Zande et al., 2012). Williams et al. (2015) observed a downregulation of genes related to the gut-associated immune response after oral administration. However, the immuno-modulating effect was solely studied in healthy animals in the absence of inflammation and great variations were observed between particle sizes, doses, and sex (Williams et al., 2015).

Further analysis should follow regarding the observed decrease in MCP-1 in the stable co-culture after AgNPs exposure, which has also been reported by Hsiao et al. (2017) in 10 nm AgNP-exposed glial cells. MCP-1 is known for its chemotactic function, but has recently been suggested to be involved in gut homeostasis, where its absence caused aggravation in a chemically-induced colitis model (Takada et al., 2010). MCP-1 is critically involved in the LPS-induced recruitment of leukocytes (Türler et al., 2002) but also responsible for limiting pro-inflammatory processes through the induction of IL-10 (Takada et al., 2010). Due to MCP-1's ambivalent role in inflammation it is unclear how an AgNP-induced reduction would affect the organism.

The similarities between AgNP- and Ag^+^-induced effects inevitably raised the question on the role of ions. AgNPs are well known for their limited stability in biological media and ample research indicates that their toxicity is proportionate to the presence of ions (Kittler et al., 2010; Smith et al., 2018). A striking example is the whole-genome expression analysis published by Bouwmeester et al. (2011). The group showed that the majority of changes induced by AgNPs with a known concentration of Ag^+^ also occurred in response to Ag^+^ alone. Our results on barrier integrity as well as the occurrence of apoptotic cells are, however, markedly different between AgNPs- and AgNO_3_-exposed cultures. Although the final toxicity might have been caused by Ag^+^, e.g. after uptake of AgNPs and subsequent lysosomal degradation, we propose that the cellular interactions might differ between ionic and particulate Ag and between stable and inflamed-like co-cultures. We do not consider the observed differences as ‘nano-specific’ effect.

## 5 Conclusions

The results showed distinct effects of AgNPs and Ag^+^ depending on the health status of the *in vitro* model at the time of exposure. In both co-culture models, AgNPs and Ag^+^ caused a significant increase in cell death with features of both apoptotic and necrotic processes. Altogether, the results suggest an increased susceptibility of inflamed compared to healthy intestinal tissue to the exposure to Ag species including AgNPs. The inflammatory potential of AgNPs remains unclear, whereas an effect on the important chemotactic protein MCP-1 cannot be dismissed. Overall, this study raises the question whether the intestinal health status inherently impacts the effects of ENM and should prospectively be considered in ENM hazard assessment.

## Ethics approval and consent to participate

Not applicable.

## Consent for publication

Not applicable.

## Availability of data and material

The datasets used and/or analysed during the current study are available from the corresponding author on reasonable request.

## Funding

Angela Kämpfer's work was supported by a PhD grant from the European Commissions' Joint Research Centre (contract number: 2012-IPR-I-20-00646).

## Authors' contributions

A.K., A.K-O and V.S. conceived and designed the experiments. A.K., P.U., R.L-S, I.O-J and N.K. performed the experiments. A.K., P.U. R.L-S, I.O-J and N.K. analysed the data. A.K. and A. K-O. wrote the paper. All contributing authors have read and approved the final version of the manuscript.

## Declaration of Competing Interest

The authors declare that they have no competing interests.

## Appendix A Supplementary data

Supplementary material Image 1
