# Supplementary material for: Ongoing inflammation enhances the toxicity of engineered nanomaterials: Application of an in vitro co-culture model of the healthy and inflamed intestine
Source: Toxicol In Vitro. 2020 Mar;63:104738. doi: 10.1016/j.tiv.2019.104738 (PMC6961208; doi:10.1016/j.tiv.2019.104738)
Supplement: Supplementary file 1 — Supplementary material [file mmc1.docx]

**Supplementary Materials**

**Ongoing inflammation enhances the toxicity of engineered nanomaterials: Application of an *in vitro* co-culture model of the healthy and inflamed intestine**

Angela A.M. Kämpfer^1,2^, Patricia Urbán^1^, Rita La Spina^1^, Isaac Ojea Jiménez^1^, Nilesh Kanase^2^, Vicki Stone^2^, Agnieszka Kinsner-Ovaskainen^1^

^1^European Commission, Joint Research Centre (JRC), Via E. Fermi 2749, 21027, Ispra (VA), Italy

^2^ Nano-Safety Research Group, School of Engineering and Physical Sciences, Heriot-Watt University, Edinburgh EH14 4AS, United Kingdom

|  |
| --- |

Fig. S1 Quantitative analysis of AgNPs in dispersant and cell culture medium based on TEM imaging

Quantitative analysis of AgNPs in dispersant (pristine) and after 4, 24, and 48h incubation in cell culture medium at 37°C, 5 % CO_2_





Fig. S2 Hydrodynamic diameter of AgNPs according to CLS

AgNPs (5 µg mL^-1^) suspended in dispersant (black line) or MEM-based cell culture medium after 4 to 48h (T4-T48 MEM) incubation at 37°C, 5 % CO_2_

| **_A_** | __ |
| --- | --- |
| **_B_** | __ |

**Fig. S3** **Quantitative analysis of DAPI-stained nuclei of Caco-2 monoculture, stable, and inflamed co-culture barriers after 24h exposure to AgNPs or AgNO_3_**

(A) Total count of nuclei and bright nuclei and (B) number of bright nuclei per 1,000 counted nuclei in Caco-2 monoculture, stable, and inflamed co-culture (CC) without exposure (control) and after 24h exposure to AgNO_3_ or AgNPs (1 or 10 μg mL^-1^) (Controls: Average ± StDev, N=3; AgNO_3_ monoculture: N=1; AgNO_3_ rest: Average ± StDev, N=2; 1 μg mL^-1^ AgNPs monoculture: N=1, rest: Average ± StDev, N=2)

| **A** |  |
| --- | --- |
| **B** |  |
| **C** |  |

Fig. S4 Relative change in the release of cytokines after 4h exposure to AgNPs or AgNO_3_ (equivalent to 1 µg mL^-1^ AgNPs) in (A) Caco-2 monoculture, (B) stable or (C) inflamed co-culture compared to the corresponding unexposed control (Average ± StDev, N=3; ^#^p≤0.05 compared to unexposed stable co-culture control; BD = below detection limit)
